# Résultats de la chirurgie avancée de la cataracte par tunnélisation: à propos de 262 cas réalisés au CHR de Banfora (Burkina Faso)

**DOI:** 10.11604/pamj.2015.22.366.8416

**Published:** 2015-12-14

**Authors:** Pierre Windinmanégdé Djiguimdé, Ibrahim Abib Diomandé, Ahgbatouhabéba Ahnoux-Zabsonré, Konan Virgile Koffi, Tierinyê Armand Meda, Gossé François Diomandé, Jerôme Sanou, Gertude Meda-Hien, Paté Sankara, Nonfounikoun Meda

**Affiliations:** 1 Service d'Ophtalmologie, CHU-YO, Ouagadougou, Burkina Faso; 2 Service d'Ophtalmologie, CHU de Bouaké, Université Alassane Ouattara, Côte d'Ivoire; 3 Service d'Ophtalmologie, CHR de Koudougou, Burkina Faso

**Keywords:** Cataracte, cécité, tunnélisation, implant, Afrique, Cataracte, cécité, tunnélisation, implant, Afrique

## Abstract

La cataracte demeure la première cause de cécité dans les pays en voie de développement posant un problème de santé publique. Le but de cette étude est de faire le bilan d'une activité de chirurgie avancée de la cataracte par la technique dite de « phacoalternative » ou par tunnélisation. Il s'agissait d'une étude prospective de base réalisée au Burkina Faso portant sur la chirurgie de la cataracte par la technique de tunnélisation. L’âge moyen de nos patients atteints de cataracte était de 62,47 ± 12,77 ans avec un sex-ratio de 0,98. La majorité des patients opérés (82,82%) étaient des cultivateurs et des ménagères. L'acuité visuelle de loin était réduite à la perception lumineuse dans 68,70% des cas. La profondeur moyenne de la chambre antérieure était de 2,98 ± 0,53 mm. L’épaisseur moyenne du cristallin était de 3,55 ± 0,75 mm. La longueur axiale moyenne de l’œil de nos patients opérés de la cataracte était de 22,88 ± 0,90 mm. La puissance réfractive moyenne estimée de la cornée était de 43,21 ± 2,03 dioptries chez nos patients. La puissance moyenne estimée de l'implant intra oculaire (IOL) utilisé était de23,11 ± 2,35D. La majorité (79,6%) de nos patients avait une bonne acuité visuelle après la chirurgie de la cataracte par tunnélisation. La chirurgie de la cataracte par tunnélisation est une technique révolutionnaire adaptée aux pays pauvres d'Afrique. Elle permet une lutte efficace à moindre coût contre la cécité par cataracte dans nos régions. La tunnélisation est une technique chirurgicale adaptée pour lutter efficacement contre la cécité due à la cataracte en Afrique.

## Introduction

Malgré l'effort consenti par l'OMS dans la lutte contre la cécité, la cataracte demeure la première cause de cécité dans les pays d'Afrique sub-saharienne avec un back log de plus de 16000 nouveaux cas /an [1]. Afin de juguler ce fléau dans le cadre de l'initiative « vision 2020 » qui stipule le droit à la vue en éliminant les cécités évitables comme la cataracte, des campagnes de chirurgie avancée sont réalisées à travers différents pays africains. Il s'agit d'une chirurgie de masse organisée dans les régions les plus reculées là où la population n'a ni les moyens financiers ni le plateau technique et le personnel qualifié pour se faire opérer. Le but de cette étude était de faire le bilan d'une activité de chirurgie avancée de la cataracte au Burkina Faso en dégageant les aspects épidémiocliniques, la valeur moyenne de l'implant utilisé et le bilan fonctionnel oculaire post opératoire.

## Méthodes

Il s'est agi d'une étude prospective de base pour la chirurgie avancée menée sur un mois dans la région des Cascades (Banfora) au Burkina Faso. Les prospections et les séances de chirurgie ont été réalisées dans la région pendant une durée de 4 semaines. La phase de sélection des patients lors de la prospection est réalisée avec toute l’équipe médicale (médecins ophtalmologistes, infirmiers spécialistes en ophtalmologie, aides-soignants) à travers les villages de la région. Elle avait pour mission principale de dépister les cataractes à opérer par un examen simple à la torche et à l'ophtalmoscope. Les différentes affections oculaires rencontrées au cours de cette phase de prospection ont fait l'objet d'une prise en charge sur place ou d'une référence vers le service d'ophtalmologie du CHR (Centre Hospitalier Régional) de Banfora. Tous les patients présentant une cécité ou une cataracte ont été convoqués au CHR la veille du début des opérations pour un examen préopératoire plus approfondi à l'issue duquel les patients présentant une cécité liée à la cataracte ont été sélectionnés et programmés pour la chirurgie. Une biométrie oculaire a été réalisée en préopératoire avec calcul de la puissance de l'implant intraoculaire emmétropisant selon la formule SRK/T afin d'optimiser les résultats fonctionnels postopératoires. Les appareils utilisés à cet effet étaient constitués de l'auto réfracto-kératomètreLuneau^®^ L66 pour la kératométrie et le biomètreTomey^®^ AL-100 pour la mesure de la longueur axiale de l’œil (LA), et le calcul de la puissance de l'implant de chambre postérieure (ICP). La biométrie a été réalisée en mode contact. Les mesures enregistrées à la biométrie étaient les valeurs moyennes après au moins une série de trois mesures. Une puissance réfractive cornéennemoyenne de 42D a été retenue pour les patients chez qui la kératométrie automatique n'a pu être réalisée faute de coopération ou du fait d'un important ptérygion. Lachirurgie a été réalisée au CHR de Banfora avec des microscopes opératoires ophtalmologiques portatifs de bonne précision et du matériel chirurgical ophtalmologique restreint. La technique chirurgicale dite chirurgie par tunnélisation ou phacoalternative (Phaco A) est une technique révolutionnaire adaptée pour l'Afrique car peu couteuse et utilisant un minimum de matériel opératoire avec des résultats satisfaisants. Le Protocole chirurgical s’établit comme suit: « Après une anesthésie locale rétrobulbaire avec 5cc de lidocaïne 2%, sur un patient prémédiqué en décubitus dorsal, une désinsertion conjonctivale au limbe est faite suivie d'une hémostase. Unepréincision d'environ 5 mm est réalisée à 3 mm du limbe. L'on procède secondairement à la réalisation d'un tunnel à partir dela préincision sclérale avec le couteau angulé 3.2 jusqu’à pénétration dans l’épaisseur cornéenne avant de procéder à une kératotomie avec reconstitution de la chambre antérieure avec du visco-élastic et élargissement de la kératotomie jusqu'aux angles irido-cornéens de part et d'autre de la pré-incision. Une capsulotomie antérieure est ensuite réalisée avec mobilisation et luxation du noyau qui est expulsé à travers le tunnel préformé. Un lavage des masses est fait suivi d'une implantation en chambre postérieure d'implant« PMMA de 6,5 mm sous visco-élastic». Les patients ont été revus à J1, J7, et J45 pour apprécier les suites postopératoires immédiates à moyen et à long terme. Les critères de l'OMS ont été utilisés pour l’évaluation des résultats fonctionnels. Ainsi, le résultat fonctionnel est jugé: bon si l'acuité visuelle est d'au moins 3/10; médiocre si l'acuité visuelle est comprise entre 1/10 inclus et 3/10 exclu; mauvais si l'acuité visuelle est strictement inférieure à 1/10. Les données recueillies ont été saisies et analysées grâce au logiciel Epi Info version 3.5.4, et le logiciel Microsoft Office 2007 a été utilisé pour certains graphiques. Pour les comparaisons de moyennes, le test paramétrique d'inégalité des moyennes de populations ANOVA a été utilisé lorsque la variance était homogène et le test de Kruskal-Wallis lorsque celle-ci n’était pas homogène. Pour le test d'inégalité des variances il a été utilisé le test de Bartlett. Les résultats ont été considérés statistiquement significatifs lorsque p (p value) était inférieur à 0,05.

## Résultats

**Figure 1:**
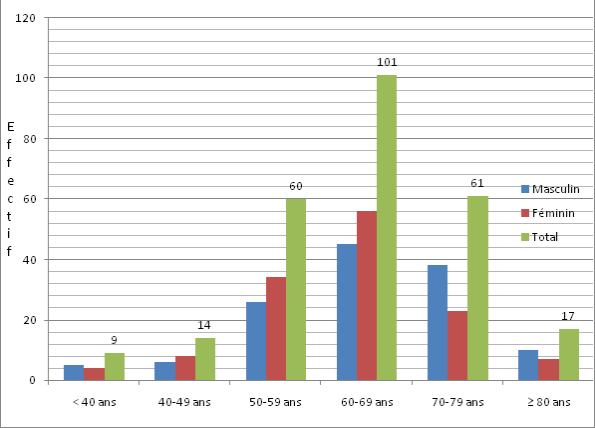
Répartition des patients atteints de cataracte selon l’âge et le sexe

**Figure 2:**
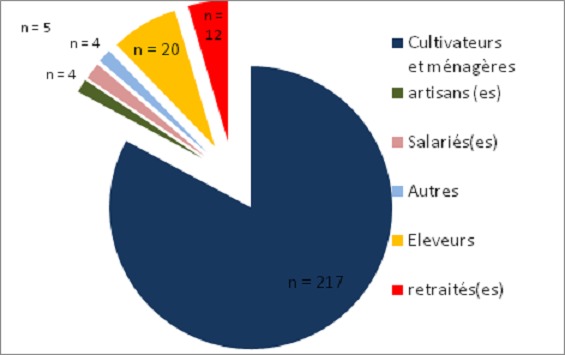
Répartition des patients atteints de cataracte selon la profession

**Figure 3:**
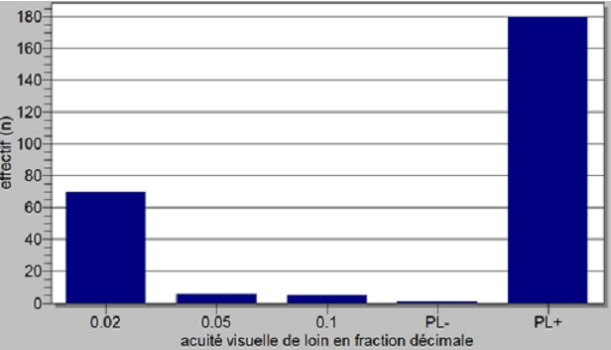
Répartition des patients atteints de cataracte selon l'acuité visuelle préopératoire

**Figure 4:**
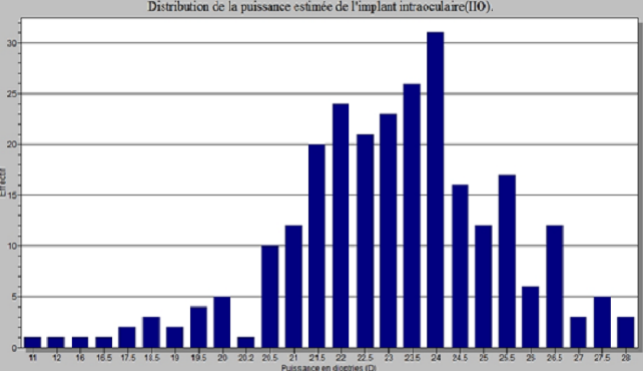
Distribution de la puissance moyenne estimée de l'implant intraoculaire

La cataracte avec 71,98% représentait la première cause de cécité chez les patients prospectés (Tableau 1). L’âge moyen de nos patients atteints de cataracte était de 62,47 ans ± 12,77 ans avec des extrêmes de 14 et de 98 ans (Figure 1). Les sujets de sexe féminin étaient les plus nombreux (50,38%) correspondant à un sexe ratio de 0,98. La majorité des patients opérés (82,82%) étaient des cultivateurs ou des ménagères (Figure 2), suivis par les éleveurs (7,63%) et les personnes à la retraite (4,58%). L'intervention concernait l’œil droit dans 52,29%. L'acuité visuelle de loin se résumait à la perception lumineuse dans 68,70% des cas (Figure 3), la meilleure acuité étant de 0,1 (1/10 ^e^). La profondeur moyenne de la chambre antérieure était de 2,98 ± 0,53 mm avec des extrêmes de 2,11 et 6,70 mm. Il n'y avait pas de différence statistiquement significative selon le genre (Tableau 2). L’épaisseur moyenne du cristallin était de 3,55 ± 0,75 mm avec des extrêmes allant de 2,13 à 7,2 mm. Il n'y avait pas de différence significative selon le genre (Tableau 3). La longueur axiale moyenne de l’œil était de 22,88 mm ± 0,90 mm avec des extrêmes de 20,92 mm et 27,70 mm. La longueur axiale moyenne de l’œil différait significativement selon le genre (Tableau 4). La puissance réfractive moyenne estimée de la cornée était de 43,21 ± 2,03 dioptries. Il existait une différence significative selon le genre (Tableau 5). La puissance moyenne estimée de l'implant intra oculaire (IOL) était de 23,11D ± 2,35D avec des extrêmes de 11 et 28D (Figure 4). La puissance moyenne de l'implant selon le sexe était de 22,59 pour le genre masculin et de 23,63 pour le genre féminin. La puissance moyenne de l'implant après réalisation de la kératométrie était de 22,58 pour le genre masculin et de 23,52 pour le genre féminin (Tableau 6). La majorité de nos patients (57,7%) avait une bonne acuité visuelle sans correction à J45 après chirurgie de la cataracte par tunnélisation (Tableau 7).

**Tableau 1:** Répartition des patients présentant une cécité selon la cause

Causes de cécité	Nombre de cas	Pourcentage
**Cataractes**	**262**	**71,98%**
Opacités cornéennes	32	8,79%
Glaucomes et neuropathies optiques	34	9,34%
Choriorétinites	16	4,39%
Traumatismes	6	1,65%
Phtise bulbaire	6	1,65%
Séquelles d'uvéites	4	1,10%
Maraboutage	2	0,55%
Ptérygion recouvrant	2	0,55%
**Total**	**364**	**100%**

**Tableau 2:** Profondeur moyenne de la chambre antérieure des patients atteints de cataracte selon le sexe

	Données valides	Moyenne (mm)	Ecart-type	Minimum	Maximum	Mode
Féminin	100	2,95	0,53	2,11	6,55	2,64
Masculin	103	3,00	0,54	2,17	6,70	2,86
Total	203	2,98	0,53	2,11	6,70	2,76
Tests	Barlett : p = 0,87			ANOVA : p = 0,39		

**Tableau 3:** Répartition des patients atteints de cataracte selon l’épaisseur moyenne du cristallin selon le sexe

	Données valides	Moyenne (mm)	Ecart type	Minimum	Maximum	Mode
Féminin	95	3,54	0,74	2,20	7,20	2,54
Masculin	97	3,56	0,61	2,13	5,19	3,62
Total	192	3,55	0,75	2,13	7,2	3,22
Tests	Bartlett : p = 0,06			ANOVA p = 0,56		

**Tableau 4:** Répartition de la longueur axiale de l’œil selon le sexe

	Données valides	Moyenne (mm)	Ecart type	Minimum	Maximum	Mode
Féminin	111	22,61	0,68	21,05	24,08	22,76
Masculin	119	23,14	0,99	20,92	27,70	21,97
Total	230	22,88	0,90	20,92	27,70	21,97
Tests	Barlett : p = 10^−4^			Kruskall-Wallis : p < 10^−3^		

**Tableau 5:** Répartition de la puissance réfractive moyenne cornéenne selon le sexe

	Données valides	Moyenne	Ecart-type	Minimum	Maximum	Mode
Féminin	124	43,58	2	38,25	52,63	42,25
Masculin	127	42,85	2	36,63	51,75	42,5
Total	251	43,21	2,03	36,63	52,63	42,5
Tests	Barlett : p = 0			ANOVA : p = 43.10^−4^		

**Tableau 6:** Répartition de la puissance moyenne estimée de l'implant intra oculaire selon le sexe chez les patients dont la kératométrie a pu être réalisée

	Données valides	Moyenne	Ecart-type	Minimum	Maximum	Mode
Masculin	127	22,58	2,42	11	28	23
Féminin	124	23,52	2,20	16	28	24
Total	251	23,11	2,35	11	28	24
Tests	Barlett : p = 0,2792			ANOVA : p = 0,0014		

**Tableau 7:** Répartition des patients selon l'acuité visuelle post opératoire à J45

	Bon	Médiocre	Mauvais	Total
Sans correction	79 (57,7%)	50 (36,5%)	8 (5,8%)	137 (100%)
Au trou sténopéique	109 (79,6%)	22 (16,0%)	6 (4,4%)	137 (100%)

## Discussion

De nombreuses pathologies oculaires sont pourvoyeuses de cécité dans les pays en développement parmi lesquelles la cataracte demeure la première étiologie avec 47% posant ainsi un problème de santé publique [2, 3]. L’âge moyen de nos patients opérés de cataracte était de 62,47 ans ± 12,77 avec des extrêmes de 14 et 98 ans. Ces données corroborent celles de la littérature confirmant qu'il s'agit d'une pathologie d’étiologies diverses pouvant survenir à tout âge [4–6]. Elle reste cependant l'apanage du sujet âgé. Les activités champêtres et domestiques étaient les principales professions pratiquées par la majorité de nos patients (82,82%). Ces chiffres sont concordants avec l'objectif de la stratégie avancée qui est d'offrir une meilleure accessibilité de la chirurgie de la cataracte aux populations démunies essentiellement rurales. L’œil droit a été l’œil le plus opéré pendant notre campagne (59,29%) il en était de même pour d'autres études [7–9]. La majorité des chirurgiens étant droitiers, leur grande aisance pour une chirurgie de l’œil droit expliquerait la chirurgie première sur l’œil droit en présence d'une cataracte bilatérale. La chirurgie de la cataracte a été réalisée en majorité chez des patients ayant une acuité visuelle réduite à la perception lumineuse.

Dans notre série, 68,7% avaient une perception lumineuse positive quand Meda [10] au Burkina et Fanny [11] en Côte-d'Ivoire trouvaient respectivement 95% et 100%. Il s'agit d'un tableau caractéristique des pays en développement où la prise en charge chirurgicale d'une cataracte est généralement retardée pour des raisons variables (pauvreté, crainte de la chirurgie, absence de chirurgien, ignorance, absence de plateau technique, inaccessibilité géographique) [12, 13]. La profondeur moyenne de la chambre antérieure avant l'intervention chirurgicale de nos patients opérés de la cataracte était de 2,98±0,53 mm avec des extrêmes de 2,11 et 6,70 mm. Il n'y avait pas de différence significative de la profondeur selon le genre (p = 0,87). Ces résultats sont superposables à ceux de Simsek et Ciftci [14] qui trouvaient en Turquie une profondeur moyenne de 2,82± 0,45 mm avec des extrêmes de 1,48 et 3,75 mm. Cependant, Salouti [15] et Chen [16] dans leurs différentes études réalisées trouvaient respectivement des profondeurs moyennes de 3,07± 0,42 et 3,12± 0,49. La différence entre ces profondeurs moyennes s'expliquerait par le fait que notre population d’étude ainsi que celle de Simsek étaient atteintes de la cataracte contrairement à celle de Salouti [15] et Chen [16]. En effet l'opacification du cristallin s'accompagnant d'une modification de sa densité, son épaisseur se trouverait affectée dans le sens d'une augmentation exerçant ainsi une pression sur la face postérieure de l'iris; ce qui serait à l'origine d'une diminution de la profondeur de la chambre antérieure ainsi que l'angle irido-cornéen. L’épaisseur moyenne du cristallin dans notre série était de 3,55 ± 0,75 mm avec des extrêmes de 2,13 et 7,2 mm. Il n'y avait pas de différence significative de l’épaisseur du cristallin selon le genre (p = 0,06). Nos résultats sont différents de ceux de Hashemi [17] en Iran et Yin [18] en chine qui trouvaient respectivement des épaisseurs moyennes de 4,28 mm et 4,56± 0,34 mm. L’épaisseur du cristallin serait-elle liée à la race?

Une étude randomisée sur différentes populations raciales serait de mise. La longueur axiale moyenne de l’œil était de 22,88± 0,90 mm avec des extrêmes de 20,92 mm et 27,70 mm. Chez le sujet masculin cette moyenne était de 23,14 mm et de 22,61 mm chez le sujet de sexe féminin (Tableau 3). Il a été noté une différence significative entre la longueur axiale de l’œil et le genre (P = 0,0001). Nos résultats corroborent ceux de Yin et Hashemi qui notaient des longueurs axiales oculaires moyennes de 23,25 ± 1,14 mm et 23,14 mm. Le genre masculin aurait donc une longueur axialeoculaire supérieure à celle du genre féminin. La puissance moyenne de l'implant intra oculaire chez nos patients chez qui la kératométrie a pu être réalisée (251 patients) était de + 23,11 ± 2,35 D avec des extrêmes de 11 et 28 D. cette puissance moyenne différait significativement selon le genre (Tableau 6). Chez les sujets masculins, la puissance moyenne était de 22,58 ± 2,42 D tandisque chez le sujet de sexe féminin, on notait une moyenne de 23,52 ± 2,20 D. S'il est admis qu'un implant intra oculaire de chambre postérieure de + 21 D devrait rendre un œil emmétrope, nos résultats semblent en être assez éloignés. Selon Aristodemou [8], la formule SRK/T serait plus indiquée pour la longueur axiale oculaire d'au moins 27 mm. Seulement un patient dans notre série avait une longueur axiale d'au moins 27 mm. Il s'en suivrait donc logiquement un biais dans l'utilisation de la formule SRK/T pour le calcul de la puissance de l'implant intra oculaire chez nos patients.

Par ailleurs, Sheard et col [19] ont développé la formule T2 après avoir constaté des aspects non physiologiques dans l'utilisation de la formule SRK/T. Ils ont conclu après une étude que l'utilisation de la formule T2 en lieu et place de la formule SRK/T améliorerait les résultats fonctionnels de 10% [19]. La chirurgie reste le seul recours pour la prise en charge de la cécité due à la cataracte. En effet,l'acuité visuelle post opératoire était améliorée chez la presque totalité de nos patients avec une majorité (79,6%) jugée bonne au trou sténopéique selon les critères de l'OMS. Ces résultats sont similaires à ceux de Maney [20] et Gogaté [21] qui trouvaient une amélioration de l'acuité visuelle après chirurgie de la cataracte par tunnélisation à des fréquences respectives de 90,98% et 98,4%. Les mauvais résultats observés dans notre étude (4,4%) étaient essentiellement liés à des complications peropératoires (rupture capsulaire) et surtout à des affections associées du segment postérieur (séquelles de choriorétinites, neuropathies optiques, etc.).

## Conclusion

La chirurgie avancée de la cataracte par tunnélisation permet une lutte accrue contre la cécité réversible due à la cataracte dans les pays en développement. Elle procure des soins de qualité à une population plus élargie tout en réduisant les coûts de leur prise en charge. L'incitation des jeunes ophtalmologistes à la pratique de la phacoalternative serait un atout remarquable dans le contexte socioéconomique de l'Afrique où la lutte contre la cécité demeure un maillon indispensable pour son développement.
